# Multifaceted characterization of the signatures and efficacy of mesenchymal stem/stromal cells in acquired aplastic anemia

**DOI:** 10.1186/s13287-020-1577-2

**Published:** 2020-02-13

**Authors:** Jiali Huo, Leisheng Zhang, Xiang Ren, Chengwen Li, Xingxin Li, Peiyuan Dong, Xuan Zheng, Jinbo Huang, Yingqi Shao, Meili Ge, Jing Zhang, Min Wang, Neng Nie, Peng Jin, Yizhou Zheng

**Affiliations:** 1 State Key Laboratory of Experimental Hematology, National Clinical Research Center for Blood Disease, Institute of Hematology & Blood Diseases Hospital, Chinese Academy of Medical Sciences & Peking Union Medical College, 288 Nanjing Road, Tianjin, 300020 China; 2 The Postdoctoral Research Station, School of Medicine, Nankai University, Tianjin, 300071 China

**Keywords:** Acquired aplastic anemia, MSCs, Biological phenotype, Genetic alterations, Immunodysregulation

## Abstract

**Background:**

Longitudinal studies have verified the pivotal role of mesenchymal stem/stromal cells (MSCs) in the bone marrow microenvironment for hematopoiesis and coordinate contribution to leukemia pathogenesis. However, the precise characteristics and alternation of MSCs during acquired aplastic anemia (AA) remain obscure.

**Methods:**

In this study, we originally collected samples from both healthy donors (HD) and AA patients to dissect the hematological changes. To systematically evaluate the biological defects of AA-derived MSCs (AA-MSCs), we analyzed alterations in cellular morphology, immunophenotype, multi-lineage differentiation, cell migration, cellular apoptosis, and chromosome karyocyte, together with the immunosuppressive effect on the activation and differentiation of lymphocytes. With the aid of whole genome sequencing and bioinformatic analysis, we try to compare the differences between AA-MSCs and HD-derived MSCs (HD-MSCs) upon the molecular genetics, especially the immune-associated gene expression pattern. In addition, the efficacy of umbilical cord-derived MSC (UC-MSC) transplantation on AA mice was evaluated by utilizing survivorship curve, histologic sections, and blood cell analyses.

**Results:**

In coincidence with the current reports, AA patients showed abnormal subsets of lymphocytes and higher contents of proinflammatory cytokines. Although with similar immunophenotype and chromosome karyotype to HD-MSCs, AA-MSCs showed distinguishable morphology and multiple distinct characteristics including genetic properties. In addition, the immunosuppressive effect on lymphocytes was significantly impaired in AA-MSCs. What is more, the cardinal symptoms of AA mice were largely rescued by systemic transplantation of UC-MSCs.

**Conclusions:**

Herein, we systematically investigated the signatures and efficacy of MSCs to dissect the alterations occurred in AA both at the cellular and molecular levels. Different from HD-MSCs, AA-MSCs exhibited multifaceted defects in biological characteristics and alterative molecular genetics in the whole genome. Our findings have provided systematic and overwhelming new evidence for the defects of AA-MSCs, together with effectiveness assessments of UC-MSCs on AA as well.

## Background

Acquired aplastic anemia (AA), a paradigm of bone marrow (BM) failure syndrome, is characterized by the absence of hematopoietic stem cells (HSCs) and the resultant pancytopenia and hypocellularity in BM [1–3]. For decades, we and other investigators have been assiduously struggling with AA in clinical practices [4, 5]. For instance, with the aid of immunosuppressive therapy (IST) and HSC transplantation, the management of acquired AA has been significantly improved [1, 6]. Unfortunately, the patients are still enduring the two major challenges including long-suffering relapse or graft failure [6]. To date, the deficiency of systematic and rigorous evaluation of the underlying mechanism severely hinders the improvement of the treatment in AA, especially the detailed alterations in hematopoietic microenvironment [7, 8].

Mesenchymal stem/stromal cells (MSCs) are heterogeneous populations capable of multipotential differentiation together with hematopoietic supporting and immunosuppressive properties [9–11]. MSCs have been wildly used in preclinical and clinical studies for disease remodeling including acute-on-chronic liver failure, diabetes, Crohn’s disease, and acquired AA [9, 12–16]. Generally, as the key component in the microenvironment, MSCs serve as a potential supplementary alternative for refractory AA treatment and have exhibited unexceptionably therapeutic effect mainly through transdifferentiation, immunomodulatory activity, autocrine, and paracrine together with providing an ideal niche [17, 18]. However, the dysfunction and pathophysiology of MSCs during acquired AA is still obscure [1, 14]. As mentioned above, our team and Hamzic et al. have identified the MSC’s involvement in the dysfunction of HSCs and immunological reconstitution in patients with AA [19, 20]. Recently, Lu et al. and Sha et al. further identified CD106 and basic fibroblastic growth factor (bFGF) were relevant with the expression of proinflammatory factors involved in AA, respectively [21, 22]. However, to our knowledge, the biofunction and molecular complexity between AA-derived MSCs (AA-MSCs) and healthy donor (HD)-derived MSCs (HD-MSCs) are incompletely understood [1, 8]. Thus, there is an urgency and necessity of conducting multifaceted comparison of MSCs from AA patients and HDs both at the cellular and molecular levels.

Herein, we systematically analyzed the abovementioned MSCs to compare their biological phenotypes and molecular genetics. In consistent with [19], MSCs in AA showed minimal differences in immunophenotype and chromosome karyotype but with sharply decreased MSC numbers when compared with those in the HDs. However, the AA-MSCs exhibited alterations in cellular morphology, cell vitality, trilineage differentiation capacity, and immunosuppressive effect as well. Different from those in the HD-MSC group, numerous distinguishable variations including global single nucleotide polymorphism (SNPs) and INDELs (insertion-deletion) signatures together with abnormal immune-associated genes were observed in the AA-MSC genome. In coincidence with the in vitro analyses, the symptoms of AA model mice were largely attenuated and the dysfunction of both hyperimmunity and pancytopenia was significantly rescued by systemic MSC transplantation.

## Methods

### Patients

Blood samples were collected from 49 acquired AA (37 severe AA and 12 non-severe AA; age, 9–69 years) patients and 39 HDs (male, 25; female, 14; age, 9–55 years). BM samples were extracted from 15 AA (9 severe AA and 6 non-severe AA; age, 13–61 years) patients and 14 HDs (male, 8; female, 6; age, 24–53 years). The characteristics of patients and HDs were listed in Additional file 11: Supplemental information, Table S1. All patients signed informed consents according to the guideline of the Declaration of Helsinki (ethics number KT2014005-EC-1). The diagnosis of AA was established in accordance with the criteria of Camitta et al [3]. All patients were newly diagnosed without definite IST at the time of sampling.

### Isolation, expansion, and identification of BM-MSCs

BM mononuclear cells (BMMNCs) were isolated using Ficoll-paque PLUS (GE Healthcare, Sweden) and cultured in Dulbecco’s modified Eagle medium: Nutrient Mixture F-12 (DME/F12; Hyclone, Logan, USA) containing 10% fetal Bovine Serum (Gibco, Thornton, Australia), 1% GlutaMAX (Gibco, Grand Island, USA), 100 U/ml penicillin/streptomycin, 2 ng/ml recombinant human basic fibroblast growth factor (bFGF; Peprotech, Rocky Hill, USA) and 10 ng/ml recombinant human epidermal growth factor (EGF; Peprotech, Rocky Hill, USA). The medium was refreshed every 3 days. After reaching 80–90% confluence, the cells were detached by TrypLE Express Enzyme (Gibco) and reseeded at a dilution of 1:3 to obtain the next generation of MSCs. The adherent cells at passage 3 were harvested to identify the phenotype of MSC with fluorescein isothiocyanate (FITC), allophycocyanin (APC), phycoerythrin (PE), PE-Cyanine 7 (PE/Cy7), or APC-Cyanine 7 (APC/Cy7)-conjugated monoclonal antibodies (mAbs): CD73, CD90, CD105, CD45, CD34, CD11b, and Human leukocyte antigen (HLA)-DR. MSCs used for subsequent functional assays were at passage 3.

### Trilineage differentiation of BM-MSCs

The differentiation capacities of MSCs into adipogenic, osteogenic, or chondrogenic lineages were performed using following kits: the MesenCult Adipogenic Differentiation kit (Stemcell, Canada), Osteogenic Differentiation kit (Gibco), or Chondrogenic Differentiation kit (Gibco) according to the manufacturer’s procedures as we previously reported [9, 11]. In brief, MSCs were seeded into 12-well plates at a density of 5 × 10^4^ cells per well and cultured until they were approximately 90–100% confluent. Then, the medium was replaced by corresponding differentiation medium and refreshed every 4 days. Oil red O staining, alizarin red S staining, and Alcian blue staining were performed to measure adipogenesis, osteogenesis, and chondrogenesis after cultured for 2 weeks, respectively. Finally, the stained cells were observed under a microscope. In addition, total RNA was extracted when the corresponding differentiation assays were completed.

### Population doubling assay

The population doubling assay was performed as we previously reported [11]. Population doubling (PD) was calculated by using the following formula PD = log_2_*N*/*N*0 in which *N*0 indicates the initial number of cells at seed and *N* represents the number of cells at harvest.

### Cell proliferation assay

The proliferation ability of BM-MSCs was measured by utilizing the Cell Counting Kit-8 (CCK-8; Dojindo, Japan). Briefly, MSCs were seeded into 96-well plates at a density of 3000 cells per well in triplications and cultured in 100 ul medium for 24, 48, 72, 96, and 120 h, respectively. CCK-8 reagents were added in a volume of 10 μl per well and incubated at 37 °C for 2 h. The absorbance of each microwell using 450 nm as the wave length was measured by microplate reader.

### Cell cycle analysis

BM-MSCs were harvested and washed with cold 1× PBS. After being fixed with 70% cold ethanol for 30 min, cells were incubated with PI/RNase staining solution (BD Pharmingen) at 4 °C for 30 min and analyzed with BD FACS Canto II system (BD Biosciences, USA).

### Apoptosis assay

The apoptosis cells were determined using PE Annexin V Apoptosis Detection Kit (BD Pharmingen) according to the manufacturer’s instructions. In brief, MSCs were harvested and washed with cold 1× PBS twice, resuspended in 200 μl binding buffer, incubated with 3 μl PE Annexin V and 5 μl 7-AAD for 15 min, and finally analyzed using flow cytometry (BD Biosciences, USA).

### Quantitative real-time PCR

Total RNA was extracted using TRIzol reagent (Invitrogen, Carlsbad, USA) as we described before [23, 24]. The reverse transcript reactions were performed using TransScript First-Strand cDNA Synthesis Supermix (TransGen Biotech, Beijing, China). Quantitative real-time PCR was performed using QuantStudio 5 system (Applied Biosystems, Carlsbad, CA) and PowerUp SYBR Master Mix (Applied Biosystems, Austin) in triplicate. The quantitation of mRNAs was calculated using the 2^−ΔΔCt^ method. The primer sequence of *ACTIN*, *ADIPOQ*, *PPAR-γ*, *RUNX2*, *ACAN*, and *SOX9* were listed in Additional file 11: Supplemental information, Table S2.

### Chromosome karyotyping

A G-banding technique was used to identify the karyotype of BM-MSCs as we previously described [11]. Chromosomes of MSCs were observed under an Olympus DP71 microscope (Tokyo, Japan) with × 200 magnification.

### The isolation of CD3^+^ T cells and coculture with BM-MSCs

Peripheral blood mononuclear cells (PBMNCs) were isolated using Ficoll-paque PLUS reagent (GE Healthcare, Sweden) as we recently reported [11]. The intracellular cytokine was measured by using the Intracellular Fixation & Permeabilization Buffer Set. In brief, cultured cells were washed with PBS once and fixed with 100 μl IC fixation buffer (eBioscience, USA) for 1 h. Then, cells were permeabilized with permeabilization buffer twice; incubated with fluorochrome-labeled mAbs: Percy5.5 anti-human CD4, PE/Cy7 anti-human CD8, FITC anti-human interferon-γ(IFN-γ), PE anti-human interleukin-4 (IL-4) and APC interleukin-17A (IL-17A); and finally analyzed by flow cytometry.

The remaining PBMNCs were used to purify CD3^+^ T cells by using positive selection CD3 microbeads following the manufacturer’s protocols (Miltenyi Biotec, Auburn, USA). For coculture analysis, MSCs were seeded onto 48-well culture plates at a density of 2 × 10^4^ cells per well and cultured overnight. After being irradiated (25 Gy), MSCs were cocultured with 2 × 10^5^ CD3^+^ T cells per well in 500 μl AIM V medium (Gibco) containing 50 ng/mL PMA (Sigma-Aldrich), 1 μg/mL ionomycin (Sigma-Aldrich), and 1 μL BD GlogiStop Protein Transport Inhibitor (BD Biosciences) for 6 h at 37 °C in 5% CO_2_. Then, cells were harvested to detect intracellular cytokine as described above. To analyze the activation of CD3^+^ T cells, irradiated MSCs were cocultured with 2 × 10^5^ CD3^+^ T cells per well in 500 μl AIM V medium (Gibco) containing 10 μg/mL anti-CD3 mAbs (Biolegend, San Diego, USA) and 2 μg/ml anti-CD28 Abs (Biolegend, San Diego, USA) for 24 h. Then, suspended T cells were harvested, washed with PBS once, and incubated with fluorochrome-labeled mAbs: Percy5.5 anti-human CD4, APC anti-human CD8, PE anti-human CD25, and FITC anti-human CD69, for 20 min at room temperature. Finally, the stained cells were analyzed by flow cytometry. The antibodies involved in this study were listed in Additional file 11: Supplemental information, Table S3.

### Immunofluorescence staining

The immunofluorescence staining was performed as we recently reported [9, 11]. Briefly, MSCs were washed three times with PBS and fixed with 3.75% formaldehyde on ice for 15 min. After being permeabilized with 0.5% Triton X-100 for 10 min, MSCs were incubated with 200 μl PBS containing 5 μl (1 U) YF-633 phalloidin (US EVERBRIGHT, Suzhou, China) at room temperature for 20 min. Then, MSCs were incubated with Hoechst 33342 (10μg/ml; Solarbio, Beijing, China) for 10 min and observed under a UltraVIEW VOX confocal microscope (Perkinelmer Inc.).

### Senescence-associated β-galactosidase assay

MSCs were seeded into 6-well plates at a density of 1 × 10^5^ cells per well for 24 h. A senescence β-galactosidase staining kit (catalog no. #9580, Cell Signaling Technology) was used to indicate the intensity of senescence according to the manufacturer’s instructions. In brief, MSCs were washed with PBS and fixed with fixative solution for 15 min at room temperature. After being washed with PBS again, MSCs were incubated with 1 ml of the β-Galactosidase Staining Solution at 37 °C overnight in a dry incubator without CO_2_ and finally observed under a microscope.

Cellular Senescence Detection Kit-SPiDER-β Gal (Donjindo, Kumamoto, Japan) was used to detect senescence in living MSCs. MSCs from AA patients and HDs were seeded into 15-mm culture dish which was precoated with 20 μg/ml Rat Tail Collagen I (Gibco, USA) for 2 h at a density of 2 × 10^4^ cells per dish. After being cultured for 24 h, MSCs were washed with culture medium once and incubated with 1 ml of Bafilomycin A1 working solution at 37 °C for 1 h in a 5% CO_2_ incubator. After adding 1 ml of SPiDER-β Gal working solution, MSCs were incubated at 37 °C for another 30 min in a 5% CO2 incubator. After removing the supernatant, MSCs were washed with 2 ml of culture medium twice and observed under a confocal microscope (Perkinelmer Inc.).

### Enzyme-linked immunosorbent assay (ELISA)

The ELISA assay was conducted as we previously described [11]. In brief, the plasma was stored at − 80 °C. The concentration of interleukin-6 (IL-6), interleukin-8 (IL-8), interleukin-10 (IL-10), tumor necrosis factor-α (TNF-α), IL-17A, and IFN-γ was measured using the corresponding ELISA kits (NeoBioscience Technology, Shenzhen, China).

### Hematoxylin and eosin (H&E) staining analysis

The analysis of H&E staining (Sigma-Aldrich) was performed according to the manufacturer’s protocols and the sections were observed under a Nikon Ti-U microscope (Nikon, Tokyo, Japan) as we reported before [9, 25].

### Cell migration

A monolayer wound-healing assay was used for detecting cell migration. After reaching 80% confluence, MSCs were scratched using a 200-μL pipette tip. At 0, 12, and 24 h, images of the scratch area were captured and analyzed by the ImageJ software.

### Induction of BM failure and UC-MSC transplantation

To induce acute AA mice, inguinal, brachial, and axillary lymph node (LN) cells from 8-week C57BL/6J mice were collected and infused into 8-week sex-matched hybrid (C57BL/6J × BALB/cBy) F1 (CByB6F1) mice at 5 × 10^6^ per recipient as previously reported with some modifications [26]. All recipients experienced a sublethal dose (5 Gy) of total body irradiation (TBI) 5 h before LN cells transfusion. Mice which received only irradiation or no intervention were considered as controls. And mice which received UC-MSC transplantation (1 × 10^6^) at day 3 of the model were regarded as the treatment group. Complete blood counts (CBCs) were detected at 0, 7, 10, and 14 days after LN cell transfusion using Sysmex’s flagship analyzer (XN-1000, USA). At day 14, mice were killed to extract BM cells, LN cells, and spleen cells. Parts of those cells were cultured in RPMI-1640 medium (Gibco) to detect intracellular cytokines as described above. Parts of these cells were collected and incubated with FITC anti-mouse CD4 and Percy5.5 anti-month CD8. All stained cells were detected by flow cytometry and analyzed by Flowjo VX software. Sternebrae were obtained for H&E staining analysis.

### Statistical analysis

Unpaired *t* test was used for analysis of two different unpaired groups, and one-way ANOVA test was used for analysis among multiple unpaired groups. All analyses were performed using Prism 6.0 (GraphPad Software, San Diego, CA, USA) as we recently described [9, 11, 23, 24]. The difference was considered significant when *P* value was less than 0.05.

## Results

### AA patients showed distinguishable immune phenotype from HDs

**Fig. 1:**
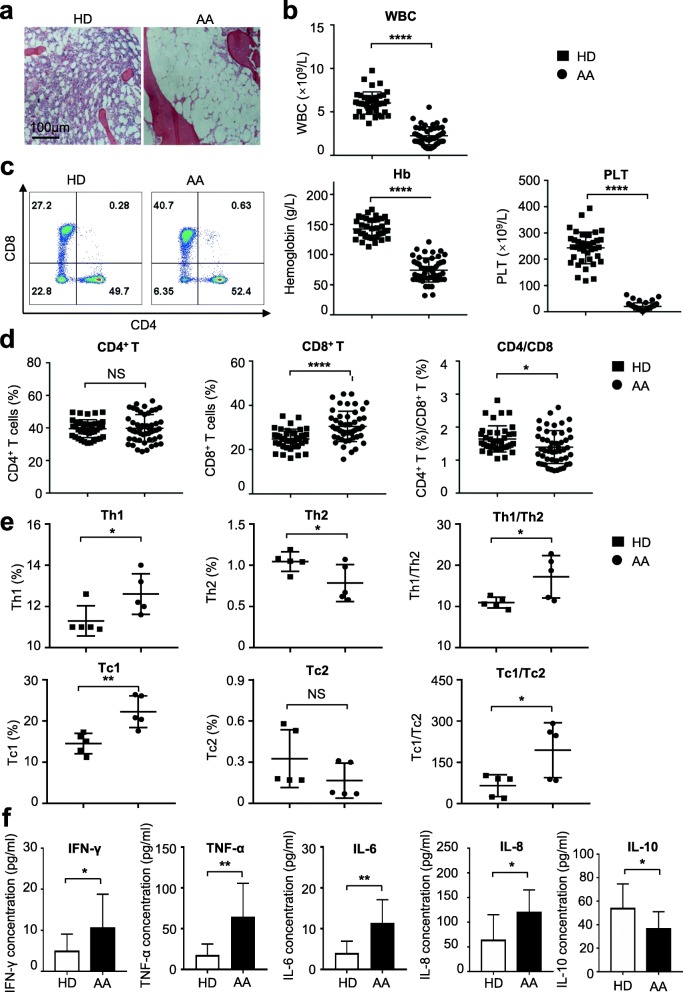
Distinguishable immune phenotypes between AA patients and HDs. **a, b** Hypocellularity in bone marrow and pancytopenia in peripheral blood was observed in AA patients. **c, d** Compared with HDs (*n* = 39), the percentage of CD8^+^ T cells was significantly increased together with markedly decreased ratio of CD4^+^ T cells to CD8^+^ T cells in AA (*n* = 49). **e** The production of interferon-γ (IFN-γ) was significantly increased in CD4^+^ T (CD4^+^IFNγ^+^IL-4^−^, Th1) cells and CD8^+^ T (CD8^+^IFNγ^+^IL-4^−^, Tc1) from AA patients (*n* = 5). Meanwhile, the ratio of Th1 to Th2 (CD4^+^IFNγ^−^IL-4^+^) and Tc1 to Tc2 (CD8^+^IFNγ^−^IL-4^+^) was also strikingly increased. **f** The concentrates of IL-6 (HD: *n* = 8; AA: *n* = 11), IL-8 (HDs: *n* = 9; AA: *n* = 12), TNF-α(HDs: *n* = 9; AA: *n* = 10), and IFN-γ(HDs: *n* = 12; AA: *n* = 16) in plasma was significantly increased in AA patients, while the concentrates of IL-10 (HDs: *n* = 9; AA: *n* = 13) were markedly decreased

To evaluate the immune status of AA, we primitively analyzed blood samples from a cohort of 49 patients with AA together with 39 HDs. The diagnosis of AA was confirmed by hypocellular BM and pancytopenia (Fig. 1a, b; Additional file 1: Figure S1a). The subsets of lymphocyte showed a higher percentage of CD8^+^ T cells in the AA group than that in HDs (Fig. 1b, c). Furthermore, the immune components including Th1 and Tc1 cells were elevated, whereas the tendency of Th2 was conversed in AA patients (Fig. 1e, Additional file 1: Figure S1b). Subsequently, the secreted cytokines in the serum were quantified. Compared with HDs, higher levels of IL-6, IL-8, TNF-α, and IFN-γbut lower concentrate of IL-10 were detected in AA patients (Fig. 1f). However, there was no difference in the concentration of IL-17A (Additional file 1: Figure S1c). Therefore, our data confirmed that AA patients exhibited a distinguishable immune phenotype from HDs. Moreover, the bone marrow samples of HD and AA patients were accurately diagnosed and appropriate for further signature and efficacy analyses of MSCs.

### AA-MSCs exhibited similar immunophenotype whereas multiple distinctions in multi-lineage differentiation

**Fig. 2:**
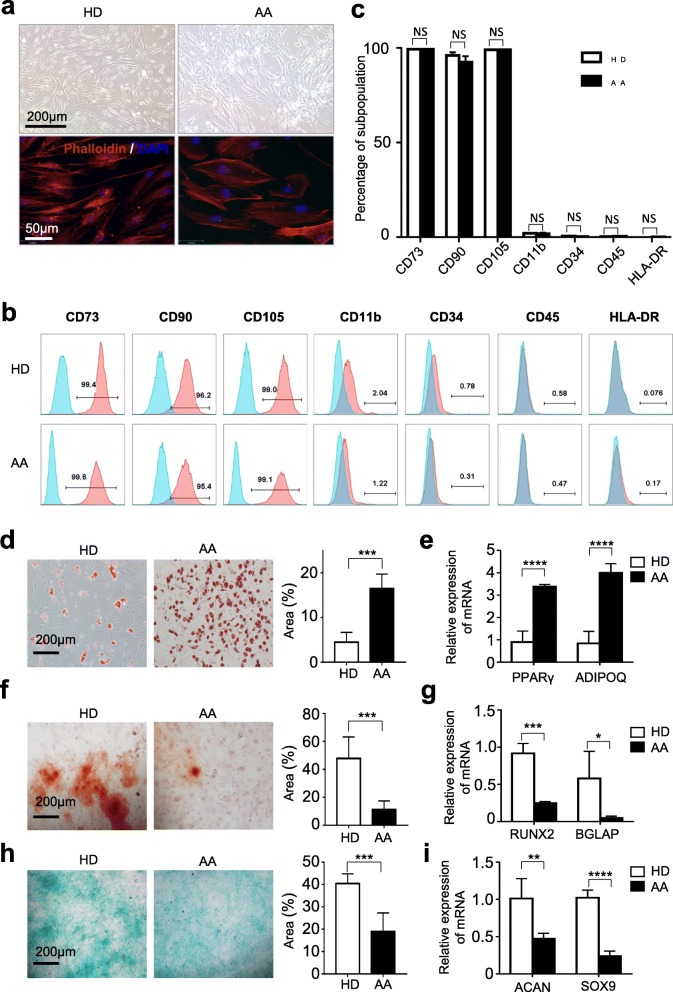
Comparisons of the morphology, identification, and tri-linage differentiation capacity of BM-MSCs between AA and HDs. **a** Distinguishable morphology of MSCs from AA and HDs. **b, c** The identification of MSCs from AA and HDs by flow cytometry (HD: *n* = 3; AA: *n* = 3). **d, e** The capacity of adipogenic differentiation by Oil Red O staining (HD: *n* = 5; AA: *n* = 5). Meanwhile, the adipogenesis-associated genes (*PPARγ* and *ADIPOQ*) were detected (HD: *n* = 4; AA: *n* = 4) by qRT-PCR. **f, g** The capacity of osteogenic differentiation by Allizarin Red staining (HD: *n* = 5; AA: *n* = 5). And the osteogenesis-associated genes (*RUNX2* and *BGLAP*) were also detected (HD: *n* = 4; AA: *n* = 4). **h, i** The chrondrogenic differentiation capacity by Alcian blue staining (HD: *n* = 5; AA: *n* = 5) and the chondrogenesis-associated genes (*ACAN* and *SOX9*) by qRT-PCR (HD: *n* = 4; AA: *n* = 4)

To systematically explore the deficiency of MSCs on AA, we successfully isolated MSCs from HDs and AA. Compared with spindle shape morphology of HD-MSCs, AA-MSCs appeared large and swollen (Fig. 2a). Immunophenotypic analysis showed that both HD-MSCs and AA-MSCs expressed high levels of mesenchymal associated biomarkers (CD73, CD90, CD105) and minimal expression of hematopoietic and endothelial associated biomarkers (CD11b, CD34, CD45) and HLA-DR (Fig. 2b, c).

Furthermore, multi-lineage differentiation analyses were conducted to clarify the potential differentiation properties. As shown by Oil Red staining, more lipid droplets were generated from AA-MSCs (Fig. 2d). And quantitative analysis of adipogenic markers showed higher levels of *ADIPOQ* and *PPAR-γ* in AA-MSCs than those in HD-MSCs (Fig. 2e). Conversely, the osteogenic differentiation potential of AA-MSCs was weaker than that in HD-MSCs, which was confirmed by both Alizarin Red staining and qRT-PCR analysis of osteogenic markers (*RUNX2* and *BGLAP*) (Fig. 2f, g). Additionally, the chondrogenic differentiation potential of AA-MSCs was weaker as well, which were verified by both Alcian Blue staining and analysis of chondrogenic markers (*SOX9* and *ACAN*) (Fig. 2h, i). Taken together, AA-MSCs showed significant differences in terms of morphology and multi-lineage differentiation capacities.

### AA-MSCs exhibited distinguishable landscape of gene expression profiling

**Fig. 3:**
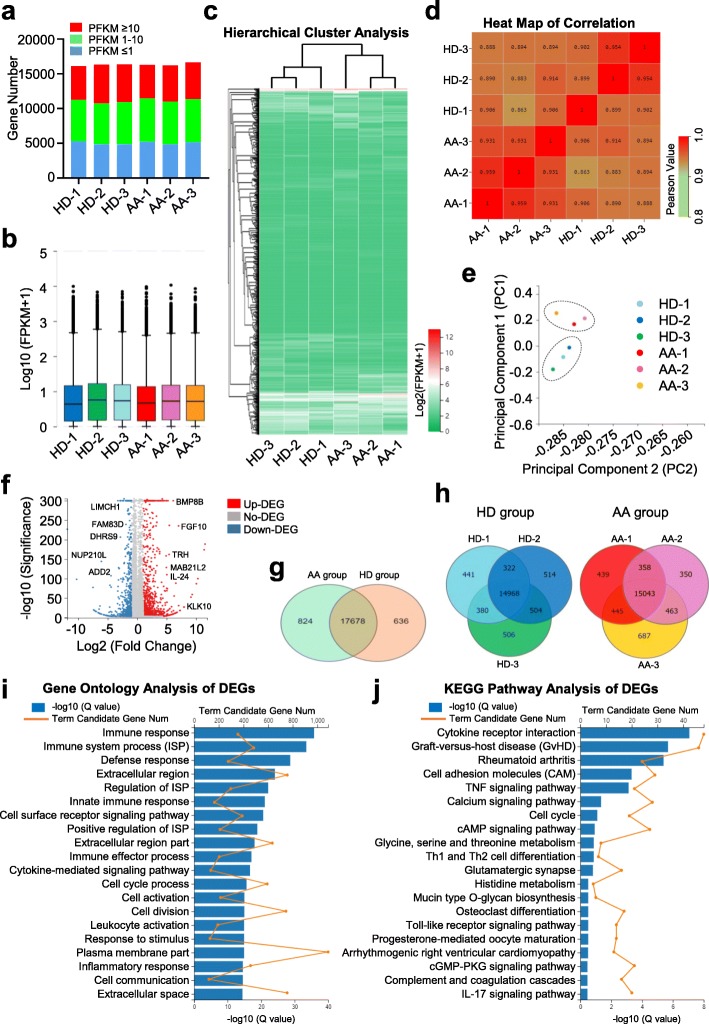
The gene expression pattern of AA-MSCs and HD-MSCs. **a, b** Similar gene expression distribution was observed in MSCs from AA (*n* = 3) and HD (*n* = 3). **c, d** Hierarchical cluster and HeatMap of correlation analysis of AA-MSCs and HD-MSCs. **e** A clear clustering between AA-MSCs and HD-MSCs was observed by principal component analysis. **f** The volcano plot analysis of AA-MSCs and HD-MSCs. **g ,h** A Venn map diagram showed the enriched genes and overlaps between AA-MSCs and HD-MSCs. **i** The gene ontology (GO) analysis showed the differentially expressed genes (DEGs) were associated with corresponding pathogenesis. **j** Different signal pathways and biological processes were enriched in AA-MSCs by KEGG pathway analysis

To further estimate the underlying molecular mechanism, we randomly and respectively selected three HD-MSCs (HD-1, HD-2, HD-3) and AA-MSCs (AA-1, AA-2, AA-3) for genome-wide RNA sequencing (RNA-seq). Primitively, all of the abovementioned MSCs showed similarity in gene expression distributions (Fig. 3a, b). However, unsupervised hierarchical clustering analysis based on fragments per kilobase per million (FPKM) values disclosed that AA-MSCs differed from HD-MSCs in evolutionary relationship, which was confirmed by the HeatMap of Pearson values (Fig. 3c, d). Furthermore, the principal component analysis (PCA) of the transcriptome showed a clear clustering between HD-MSCs and AA-MSCs (Fig. 3e).

To gain further insights into the differences between the two groups, we conducted volcano plot analysis and found that numerous differentially expressed genes were enriched in HD-MSCs (ADD2, DHRS9, etc.) or AA-MSCs (BMP8B, IL-24, etc.), respectively (Fig. 3f, Additional file 2: Figure S2a, b). With the aid of a Venn map diagram, a total number of 19,138 differentially expressed genes (DEGs) were clustered into three different categories with 17,678 overlaps between HD-MSCs and AA-MSCs (Fig. 3g, Additional file 6: Table S1). Additionally, we also noticed that a total number of 14,968 and 15,043 genes were highly enriched in the HDs and AA groups, respectively (Fig. 3h). To elucidate the potent influence of the DEGs to AA-associated pathogenesis, we took advantages of the gene ontology (GO) analysis and found that the upregulated DEGs were principally associated with immunoregulation (e.g., immune response, immune system process) and cellular process (e.g., cell cycle process, cell division) (Fig. 3i). Accordingly, immunologically relevant signaling pathways (TNF, toll-like receptor (TLR), IL-17, etc.) and biological process (graft-versus-host disease (GvHD), cell adhesion molecules (CAM), cell cycle and differentiation, etc.) were enriched as well by utilizing Kyoto Encyclopedia of Genes and Genomes (KEGG) pathway analysis (Fig. 3j). Taken together, with the aid of bioinformatics, we explored the differential gene expression pattern together with the underlying influences to the biological phenotype and functional deficiency of AA-MSCs.

### Multiple genetic mutations and variation spectrums were enriched in the chromosome of AA-MSCs

**Fig. 4:**
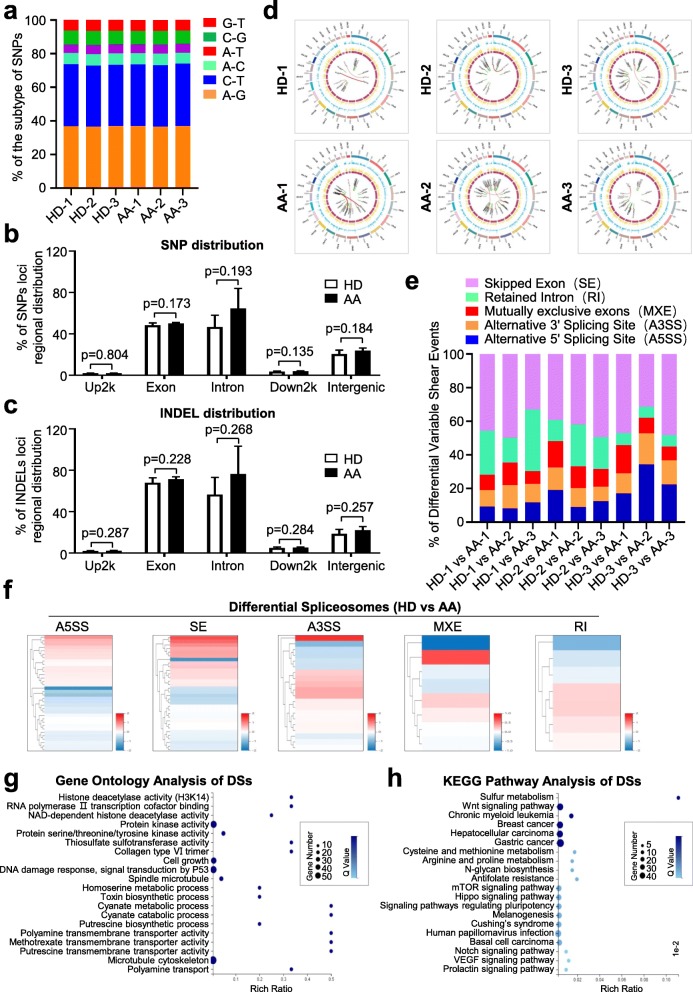
Gene mutations and variation spectrums in the chromosome of AA-MSCs and HD-MSCs. **a–c** There was no significant difference in the subtypes of single nucleotide polymorphisms (SNPs), SNP distribution and insertion/deletion (INDEL) distribution between AA-MSCs and HD-MSCs. **d** Different somatic variations and gene fusion events were analyzed by Circos software. And their loci regional distribution was also further confirmed. **e, f** Differential variable shear events (DVSE) including skipped exon (SE), retained intron (RI), mutually exclusive exon (MXE), alternative 3′ splicing site (A3SS), and alternative 5′ splicing site (A5SS) were analyzed. And SE accounts for 58% of DVSE. **g, h** The GO analysis showed differential spliceosomes (DSs) were relevant to a series of biological process. **h** Enriched DSs were associated with different signaling pathways

To further estimate the potential molecular abnormalities, we compared AA-MSCs with HD-MSCs from the perspective of genetic modification including single nucleotide polymorphisms (SNPs), insertion-deletion (INDELs), and spliceosomes. Primitively, based on the distribution of the 75,374~128,557 SNPs in the whole genome, none of the five subtypes of SNPs showed significant statistical differences in HD-MSCs and AA-MSCs (Fig. 4a, b, Additional file 3: Figure S3a, Additional file 7: Table S2). Simultaneously, with the aid of the genome analysis toolkit (GAT) [27], we did not find visible differences between the two groups as well (Fig. 4c, Additional file 3: Figure S3b). The somatic variation analysis of SNPs, INDELs, FPKMs, and gene fusion events (e.g., ATP5I-AP3D1, BLOC1S1-RDH5, CLCF1-POLD4, ACCS-EXT2) by the Circos software further conformed their loci regional distribution and expression in the chromosome (Fig. 4d, Additional file 3: Figure S3c). In addition, we conducted statistical analysis of spliceosomes based on the differential variable shear events (DVSE) including skipped exon (SE), retained intron (RI), mutually exclusive exon (MXE), alternative 3′ splicing site (A3SS), and alternative 5′ splicing site (A5SS). Of the DVSE events, we noticed that SE was the major subtype in the vicinity of 58% (Fig. 4e, f; Additional file 8: Table S3). Moreover, by conducting GO analysis, we found that the abovementioned differential spliceosomes (DSs) were relevant to a series of epigenetic modifications (e.g., histone deacetylase activity) and cellular process (e.g., cell growth, metabolic process) (Fig. 4g). Similarly, the enriched DSs were related to oncogenesis, metabolism, and signaling pathways including Wnt, mTOR, Hippo, Notch, and VEGF (Fig. 4h).

### AA-MSCs showed deficiency in multiple cytological signatures

**Fig. 5:**
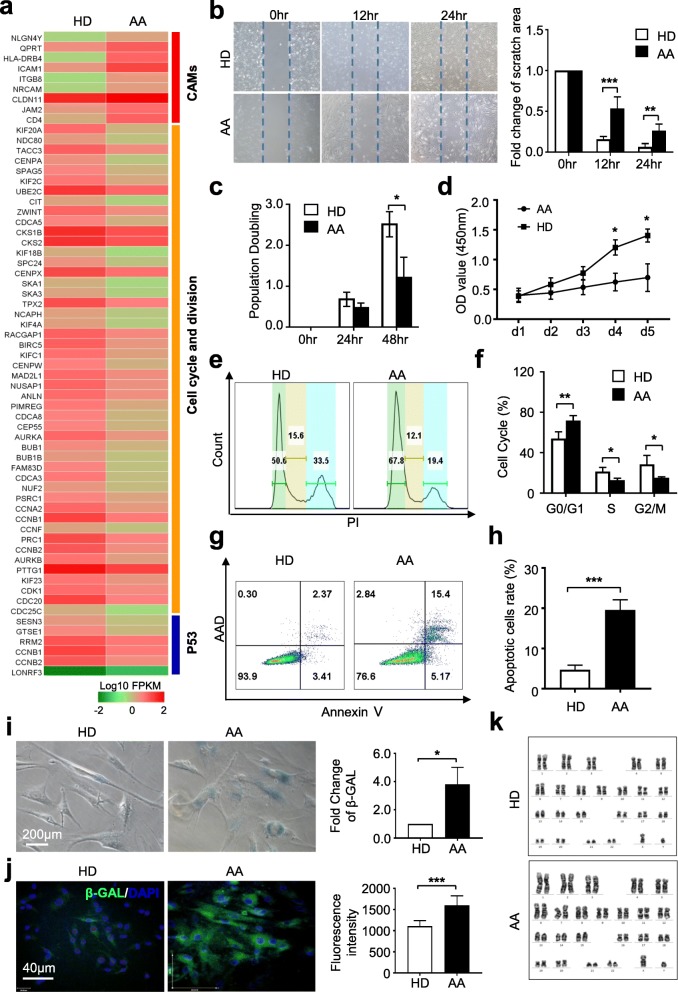
The intrinsic deficiency of AA-MSCs. **a** Multiple genes associated with cell adhesion molecules (CAMs) were highly expressed in AA-MSCs, while cell cycle- and p53-associated genes were decreased in AA-MSCs. **b** The scratch analysis showed a decreased migration capacity of AA-MSCs (HD: *n* = 5; AA: *n* = 5). **c, d** The proliferative capacity of AA-MSCs was significantly decreased (HD: *n* = 5; AA: *n* = 5). **e, f** The cell cycle analysis showed a higher proportion in sub-G1 phase in AA-MSCs (HD: *n* = 8; AA: *n* = 8). **g, h** Significantly increased apoptotic cells was measured in AA-MSC by flow cytometry (HD: *n* = 6; AA: *n* = 6). **i, j** Increased cell senescence was observed in AA-MSCs by using β-GAL staining under light microscope and confocal microscope (HD: *n* = 5; AA: *n* = 5). **k** No karyotype abnormalities were observed in AA-MSCs (HD: *n* = 3; AA: *n* = 3)

Having disclosed the genetic characteristics between HD-MSCs and AA-MSCs, we wondered whether the biological properties were also different. As shown by the clustering diagram, cell adhesion molecule (CAM)-associated genes were highly expressed while the cell cycle and division- and P53-associated genes were declined in AA-MSCs (Fig. 5a). Expectedly, inconsistent with the gene expression pattern of CAMs, the migration capacity of AA-MSCs was impaired when compared with HD-MSCs (Fig. 5b). We subsequently examined the long-term proliferation ability of MSCs and found that the population doubling (at 48 h) and cell proliferation of AA-MSCs (at day 4 and day 5) were both significantly declined (Fig. 5c, d). Furthermore, by flow cytometry analysis, we revealed the different cell cycle phase distributions of AA-MSCs from those of HD-MSCs (Fig. 5e, f). For example, AA-MSCs were characterized by a higher percentage of populations in the sub-G1 phase of cell cycling, which indicated an increasing proportion of apoptosis in AA-MSCs (Fig. 5f). In coincidence, the percentage of apoptotic cells in HD-MSCs was nearly threefold of that in HD-MSCs (Fig. 5g, h). In addition, a higher proportion of cells with senescent properties in AA-MSCs were probed by β-GAL staining (Fig. 5i, j). In contrast, AA-MSCs exhibited normal karyotype without gross abnormalities at the genomic level as HD-MSCs, which was identified by the G-banded chromosome analysis (Fig. 5k).

### Multi-dimensional analyses identified a distinguishable immune signature in AA-MSCs

**Fig. 6:**
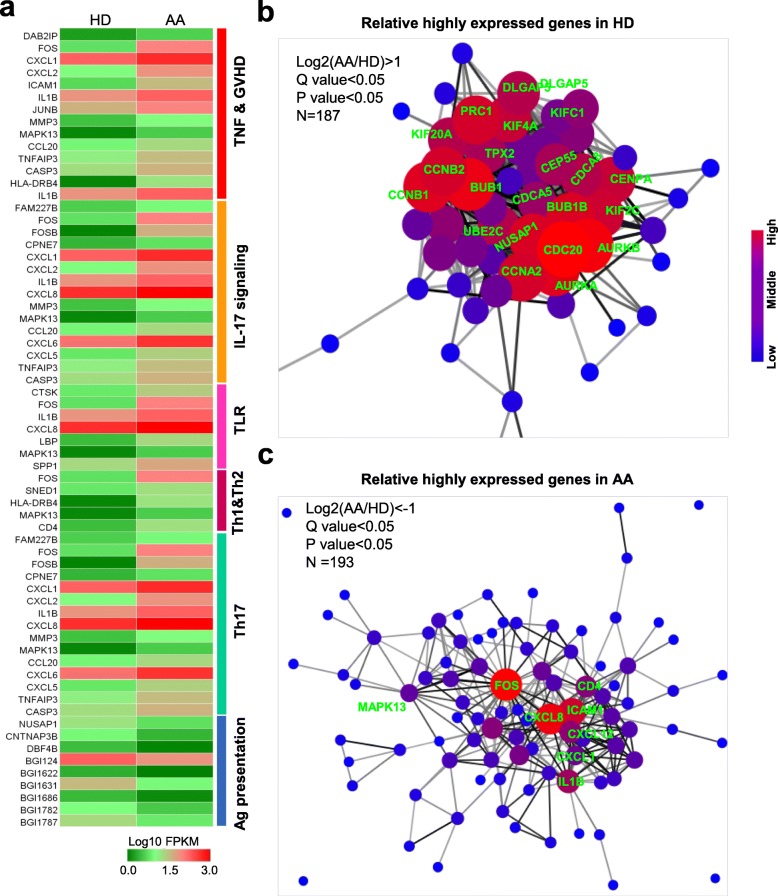
Distinguishable immune signature in AA-MSCs. **a** The immune-associated gene sets in AA-MSCs. Relative highly expressed genes in **b** HD-MSCs and **c** AA-MSCs

To further illuminate the potential AA-MSC alterations, we compared immunoregulatory associated gene sets with HD-MSCs. Among them, we noticed the enriched and upregulated genes in the subsets of TNF and GVHD, IL-17 signaling, and TLR (e.g., FOS, IL1B, CXCLs) whereas downregulated antigen presentation-associated genes (e.g., NUSAP1, CNTNAP3B) in AA-MSCs. In addition, Th1, Th2, and Th17 differentiation-associated genes were also highly expression in AA-MSCs as shown by the HeatMap diagram (Fig. 6a). Furthermore, protein interaction analyses by utilizing the DIAMOND and STRING database indicated the distinct pivotal proteins between HD-MSCs and AA-MSCs. As shown in Fig. 6b, numerous genes-involved in innate immune system and promoting abnormal protein degradation (KIFs, UBE2C), cell division and cytokinesis (CCNs, CDCs, AURKs, CEPs, NUSAP1), protein kinase (BUBs), and even alternative splicing process (PRC1) were highly expressed in HD-MSCs (Additional file 9: Table S4). In contrast, the pivotal inflammation-related genes (FOS, CXCL8, etc.) were highly expressed and formed a tightly connected network, which indicated the abnormal gene pattern of AA-MSCs (Fig. 6c, Additional file 10: Table S5). These results clearly displayed complex and multi-layered alterations of AA-MSCs in terms of whole-genomic status when compared to HD-MSCs.

**Fig. 7:**
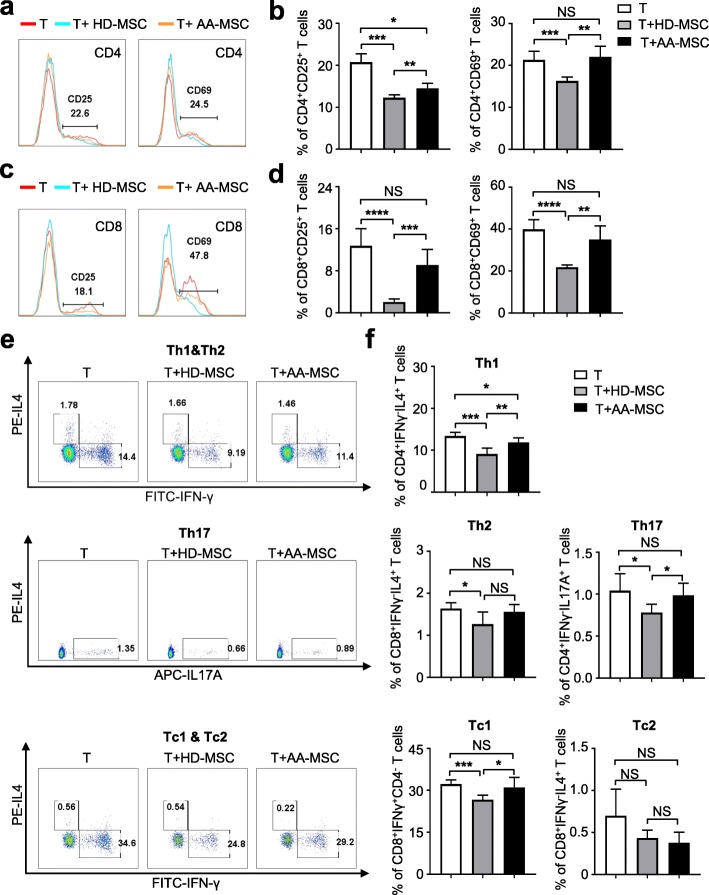
Impaired immune inhibition of AA-MSCs. **a–d** Compared with HD-MSCs, AA-MSCs showed a defected capacity to inhibit the activation of CD4^+^ and CD8^+^ T cells (HD: *n* = 5; AA: *n* = 5). **e**, **f** The capacity to inhibit T cells towards Th1 (CD4^+^IFNγ^+^IL4^−^), Th17 (CD4^+^IL4^−^IL17A^+^), and Tc1 (CD8^+^IFNγ^+^IL4^−^) were markedly decreased in AA-MSCs (HD: *n* = 5; AA: *n* = 5), while there was no difference in inhibiting T cells into Th2 (CD4^+^IFNγ^−^IL4^+^) and Tc2 (CD8^+^IFNγ^−^IL4^+^) between AA-MSCs and HD-MSCs

Previously, we and other investigators have demonstrated that adult MSCs could inhibit the proliferation of peripheral blood mononuclear cells (PBMCs) and mixed lymphocytes. Herein, we took advantage of the model to explore whether AA-MSCs had any defect in immunoregulation. Compared to the HD-MSCs group, the inhibitory capacities of AA-MSCs on activation of CD3^+^ T cells and differentiation towards Th1 and Tc1 were strikingly impaired (Fig. 7a–f). Taken together, our results implicated the molecular abnormality and immunoregulation impairment of MSCs in AA patients.

### UC-MSC transplantation effectively ameliorated the symptoms of AA mice

**Fig. 8:**
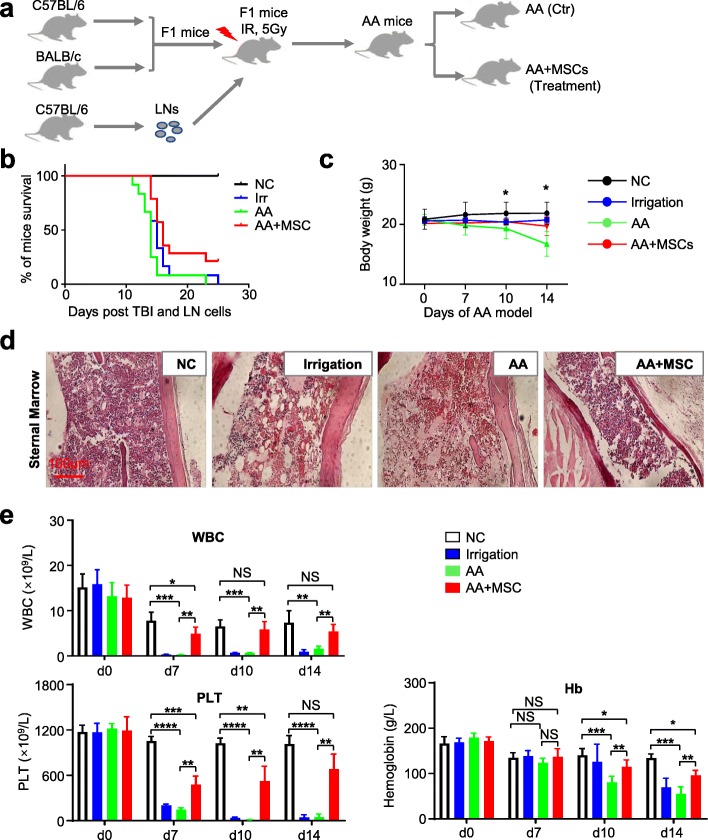
UC-MSC transplantation effectively ameliorated the symptoms of AA mice. **a** The model of AA mice. **b, c** AA mice receiving UC-MSC transplantation showed reduced mortality and prolonged survival, along with alleviating body weight decline (normal mice: *n* = 5; irrigation mice: *n* = 5; AA mice: *n* = 5; AA+MSC: *n* = 5). **d** The hypocellularity of bone marrow in AA mice was rescued after UC-MSC transplantation (normal mice: *n* = 3; irrigation mice: *n* = 3; AA mice: *n* = 3; AA+MSC: *n* = 3). **e** Increased peripheral blood cell count including white blood cell (WBC), hemoglobulin, platelets (PLT), neutrophil cell, reticulocyte, and red blood cell (RBC) were observed in AA mice after receiving UC-MSC transplantation (normal mice: *n* = 3; irrigation mice: *n* = 3; AA mice: *n* = 3; AA+MSC: *n* = 3)

Having illuminated the potential role of AA-MSCs on AA-associated pathogenesis both at the cellular and molecular level, we next aimed to explore whether MSCs were a pivotal microenvironmental component for the treatment of AA. Herein, we took advantage of a recently described acute aplastic model to assess whether AA mice could be effectively ameliorated (Fig. 8a). Compared with the control mice (AA mice with 1 × PBS injection), AA mice which received systemic infusion of UC-MSCs (1 × 10^6^ UC-MSCs, intravenous injection at day 3 of the model) exhibited reduced mortality and prolonged survival, together with alleviating the body weight decline (Fig. 8b, c). Consistent with the observation of general signs, AA mice which received UC-MSC injection showed significantly alleviated AA-associated pathological features of sternum (Fig. 8d).

**Fig. 9:**
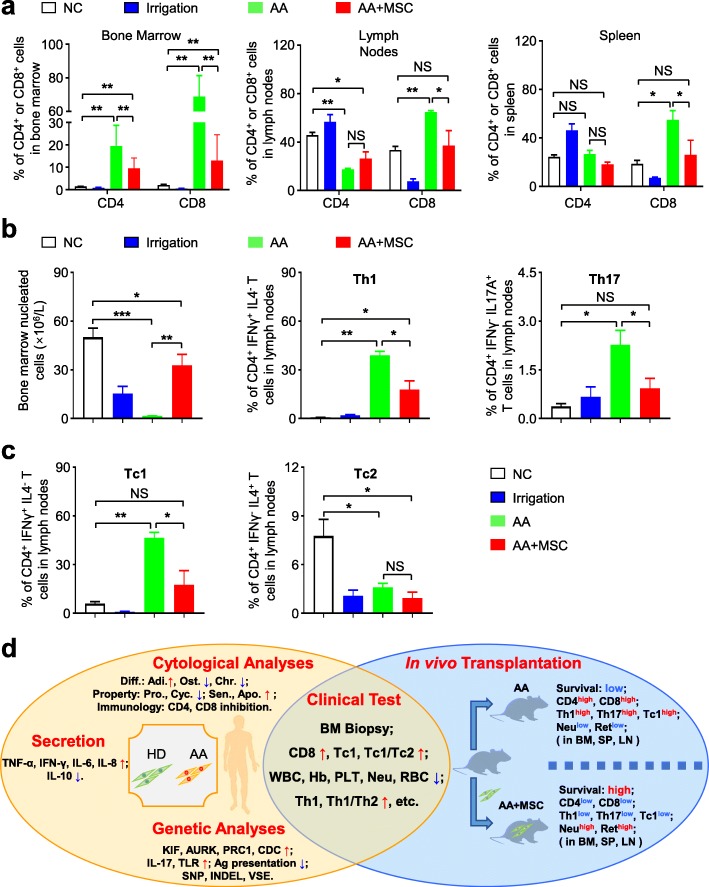
Effective immunoregulatory capacity of UC-MSCs in AA mice. **a** After receiving UC-MSC transplantation, AA mice had a decreased proportion of CD4^+^ and CD8^+^ T cells in bone marrow and spleen, while, in lymph nodes, only CD8^+^ T cells was decreased (normal mice: *n* = 3; irrigation mice: *n* = 3; AA mice: *n* = 3; AA+MSC: *n* = 3). **b**, **c** UC-MSC transplantation increased the bone marrow nucleated cells in AA mice (normal mice: *n* = 3; irrigation mice: *n* = 3; AA mice: *n* = 3; AA+MSC: *n* = 3). Additionally, Th1, Tc1, and Th17 cells were significantly decreased in AA mice after infusing UC-MSCs. Meanwhile, the ration of Th1 to Th2 and Tc1 to Tc2 were also markedly decreased, while the proportions of Th2 and Tc2 were not remarkably influenced (normal mice: *n* = 3; irrigation mice: *n* = 3; AA mice: *n* = 3; AA+MSC: *n* = 3). **d** Intrinsic impairment of MSCs and abnormal immune status were detected in AA patients. Furthermore, UC-MSC transplantation could effectively ameliorate the hyperimmune status and restore hematopoiesis

Routine analysis of peripheral blood further indicated that UC-MSC transplantation could effectively ameliorate pancytopenia and restore blood parameters, including the number of white blood cells (WBC), hemoglobin (Hb), platelets (PLT), neutrophil, reticulocyte, and red blood cells (RBC) compared with mice in the irrigated and AA groups (Fig. 8e, Additional file 4: Figure S4a–c). Meanwhile, mice received UC-MSC infusion also regained immunoregulatory capacity to some extent, for the proportions of CD4^+^ and CD8^+^ T lymphocytes were evidently declined in BM, lymph nodes, and spleen (Fig. 9a). To systematically evaluate the therapeutic effect of UC-MSCs on AA mice, we conducted flow cytometry analysis of immunocytes in mice. At day 14, the total number of BMNCs, together with immunocytes including Th1, Th2, Th17, Tc1, and Tc2, in the UC-MSCs group were close to normal levels (Fig. 9b, c; Additional file 5: Figure S5a, b). Collectively, systemic administration of UC-MSCs could significantly ameliorate the pathological damages and regain BM hematopoiesis and immunoregulatory capacity of mice with AA.

## Discussion

Although the remarkable improvements had been achieved in the management of AA following IST, up to 30% of patients relapsed [28, 29]. Additionally, patients who receiving matched sibling donor hematopoietic cell transplantation could still experience graft failure [6]. Hence, BM microenvironment which might contribute to the abnormal immune and impaired hematopoietic supporting arouse our attention. In the year of 2012, we originally reported several defects of AA-MSCs including abnormal morphology, declined proliferation and increased apoptosis, which was further confirmed by Hamzic et al. [19, 20]. However, the multifaceted formulation of pivotal defects of AA-MSCs both at the cellular and molecular levels is largely unknown. In this study, we systematically evaluated the biological and genetic signatures of human AA-MSCs, together with the therapeutic effect of MSC transplantations on AA mice. In spite of the scarcely visible differences in immunophenotypes, AA-MSCs showed more distinguishable alterations both at the cellular and molecular levels. Together with the in vivo transplantation analysis, our data provided an overwhelming evidence for MSCs as a pivotal microenvironment component, which could help understand the pathogenesis of AA (Fig. 9d).

Currently, the interaction between AA and bone marrow microenvironment has become a focus of the field. As the key component of the hematopoietic niche, BM-MSCs insufficiency would contribute to leukemia pathogenesis and even result in niche alterations and hematopoietic impairment in AA patients [30, 31]. Previously, Wu and colleagues found that BM-MSCs in severe AA had defect in suppressing the proliferation of PBMNCs but with increased apoptosis [31]. Recently, we and other investigators further confirmed the alternation in multiple cytokine secretion and the defects in immunosuppressive capacity of AA-MSCs. However, the current studies on AA-associated MSCs are still piecemeal and the systematically cognitive explanation of the pathogenesis is woefully inadequate as well. Herein, at the cellular level, we have thoroughly demonstrated that the MSCs in AA patients exhibited multifaceted alterations including the abnormal morphology, decreased population doubling and cell cycle, and impaired multi-lineage differentiation and immunoregulatory capacity together with deteriorative apoptosis and senescence.

In coincidence with the phenotypic signatures, multi-dimensional analyses of genome-wide RNA profiling revealed the distinguishable landscape of gene expression pattern and multiple genetic variation spectrums in the chromosome of AA-MSCs. On the one hand, different from those in the HD-MSCs group, AA-MSCs highly expressed proinflammatory and cancer-associated genes (IL1B, IL-24, CXCLs, FOS, KLK10). By contrast, proliferation- and immunoregulation-associated genes (CDCs, CCNs, KIFs, AURKs, PRC1) were downregulated in AA-MSCs. What is worse, the DEGs involved in multiple signals (TNF, cAMP, TLR, IL-17) resulted in the reduction in immune response, leukocyte activation, and cell division in AA-MSCs. On the other hand, the alterations in SNP and INDEL distribution and gene fusion could affect the epigenetic and metabolic process by activating a series of cancer-associated cascades (Wnt, mTOR, Hippo, Notch, VEGF). Above all, the interactive pattern of DEGs in AA-MSCs showed multiple distinctions from that in the HD-MSCs, especially the immunosuppression and cell viability-associated genes. Thus, in conjunction with the aforementioned in vivo transplantation data, we have provided systematic and overwhelming evidence for illuminating the pivotal characteristics and pathogenesis of MSCs in AA. Overall, our study offers new references for investigating pathogenic mechanism of AA.

## Conclusions

Overall, distinguished from HD-MSCs, numbers of categorical variables and defects were observed in AA-MSCs. In this study, we have uncovered the landscape of the intrinsic attributes and provided new overwhelming evidence of MSCs in AA treatment.

## Supplementary information

**Additional file 1: Figure S1.** The characteristics of AA patients.

**Additional file 2: Figure S2.** The Heatmap of differentially expressed genes between AA and HD.

**Additional file 3: Figure S3.** The enriched genetic mutations and variation spectrums in the chromosome of AA-MSCs.

**Additional file 4: Figure S4.** UC-MSC transplantation ameliorate the pancytopenia in AA mice.

**Additional file 5: Figure S5.** UC-MSC transplantation significantly rescue the hyperimmune status of AA mice.

**Additional file 6: Table S1.** Total number of differentially expressed genes (DEGs)

**Additional file 7: Table S2.** The SNP variations of AA-MSCs and HD-MSCs.

**Additional file 8: Table S3.** The numbers of variable shear events in AA-MSCs and HD-MSCs.

**Additional file 9: Table S4.** The up-regulated genes in AA-MSCs.

**Additional file 10: Table S5.** The down-regulated genes in HD-MSCs.

**Additional file 11: Supplementary information.** The details associated with Additional Figure Legends and Additional Tables were listed.

## Data Availability

All data generated or analyzed during this study are included in this published article and its supplementary information files. Meanwhile, the datasets used and analyzed during the current study are also available from the corresponding author on reasonable request.

## Abbreviations

MSCs: Mesenchymal stem/stromal cells
AA: Aplastic anemia
HD: Healthy donor
UC-MSC: Umbilical cord-derived MSC
BM: Bone marrow
HSC: Hematopoietic stem cell
IST: Immunosuppressive therapy
AA-MSCs: AA-derived MSCs
HD-MSCs: HD-derived MSCs
SNPs: Single nucleotide polymorphism
INDELs: Insertion/deletion
BMMNC: Bone marrow mononuclear cell
ELISA: Enzyme-linked immunosorbent assay
H&E: Hematoxylin and eosin
TBI: Total body irradiation
FPKM: Fragments per kilobase per million
PCA: Principal component analysis
DEGs: Differentially expressed genes
GO: Gene ontology
GVHD: Graft-versus-host disease
KEGG: Kyoto Encyclopedia of Genes and Genomes
GAT: Genome analysis toolkit
DVSE: Differential vatable shear events
CAM: Cell adhesion molecules
