# Supplementary figures and images for: Multifaceted characterization of the signatures and efficacy of mesenchymal stem/stromal cells in acquired aplastic anemia
Source: Stem Cell Res Ther. 2020 Feb 13;11:59. doi: 10.1186/s13287-020-1577-2 (PMC7020384; doi:10.1186/s13287-020-1577-2)

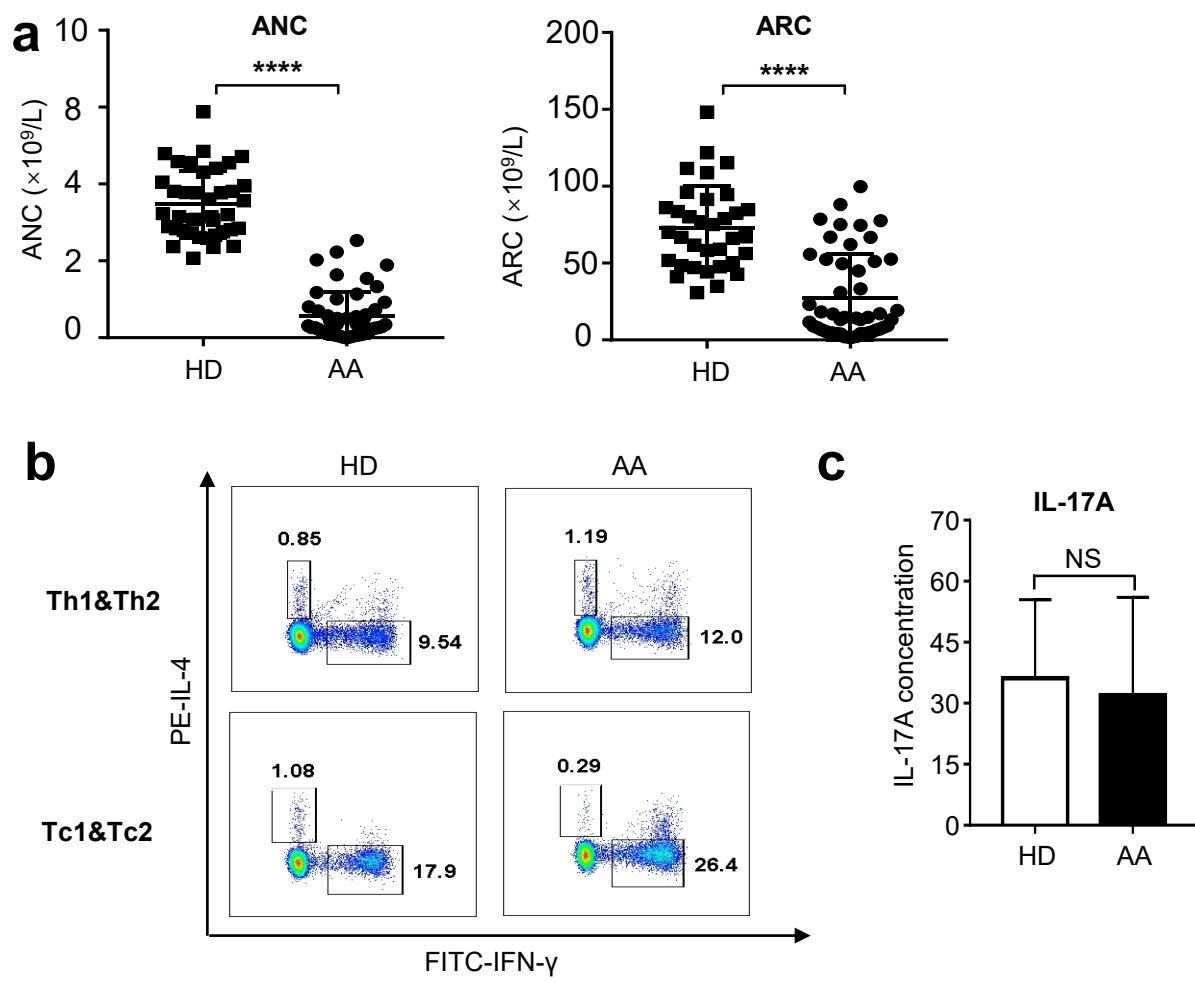

Figure S1

Supplement: Supplementary file 1 — Additional file 1: Figure S1. The characteristics of AA patients. [file 13287_2020_1577_MOESM1_ESM.pdf]

**a**

Up-regulated genes (AA vs HD)

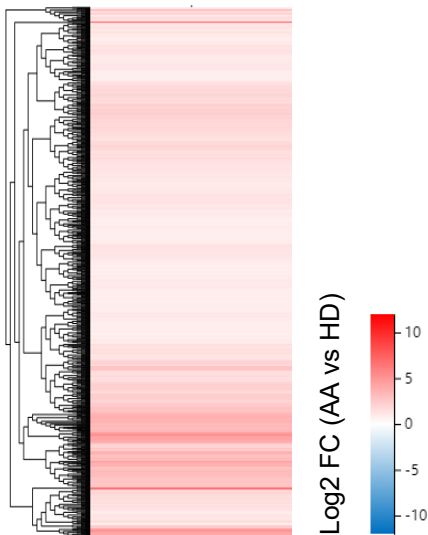

**b**

Down-regulated genes (AA vs HD)

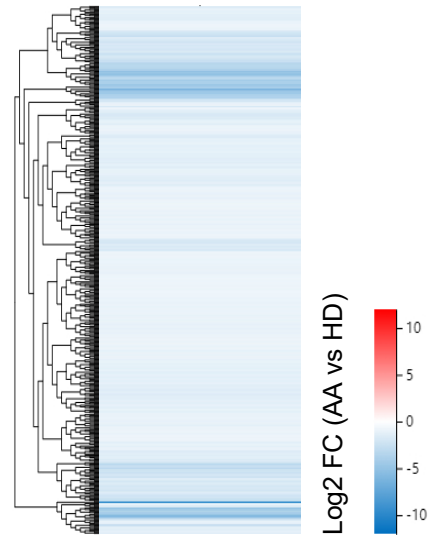

Supplement: Supplementary file 2 — Additional file 2: Figure S2. The Heatmap of differentially expressed genes between AA and HD. [file 13287_2020_1577_MOESM2_ESM.pdf]

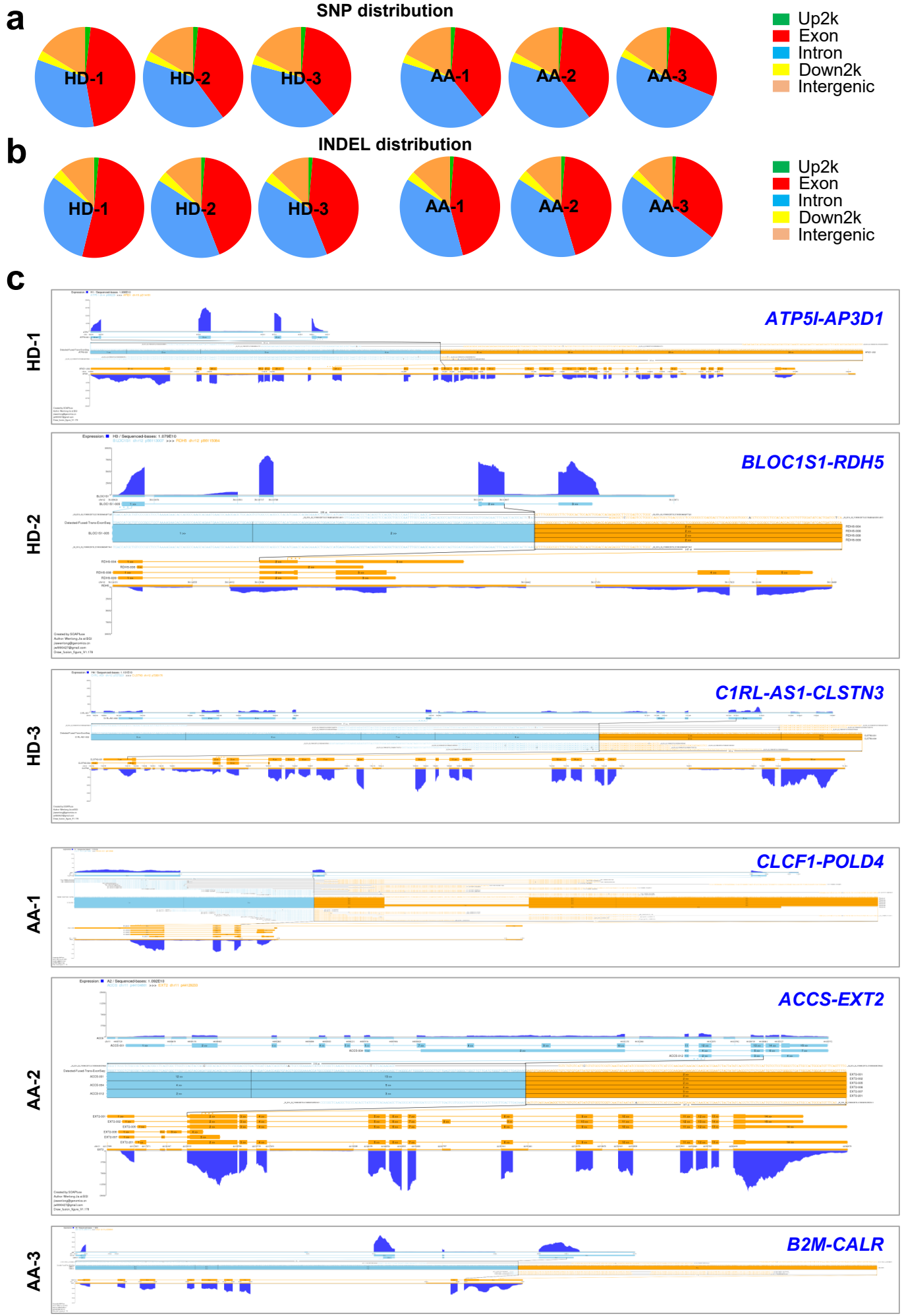

Supplement: Supplementary file 3 — Additional file 3: Figure S3. The enriched genetic mutations and variation spectrums in the chromosome of AA-MSCs. [file 13287_2020_1577_MOESM3_ESM.pdf]

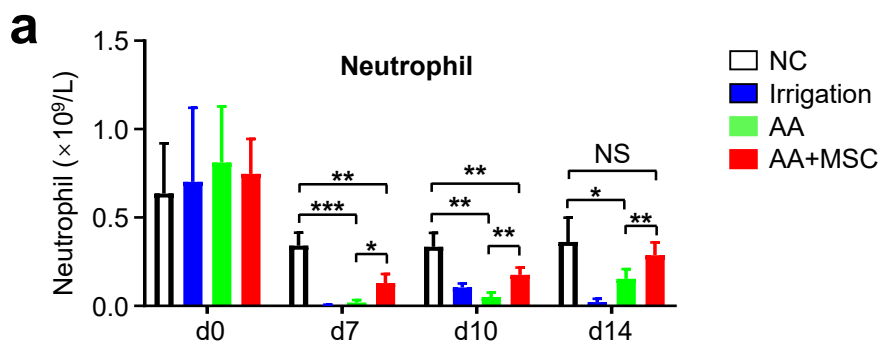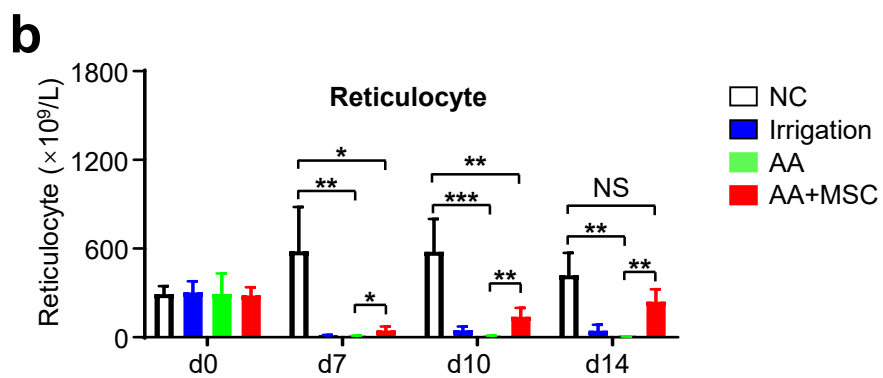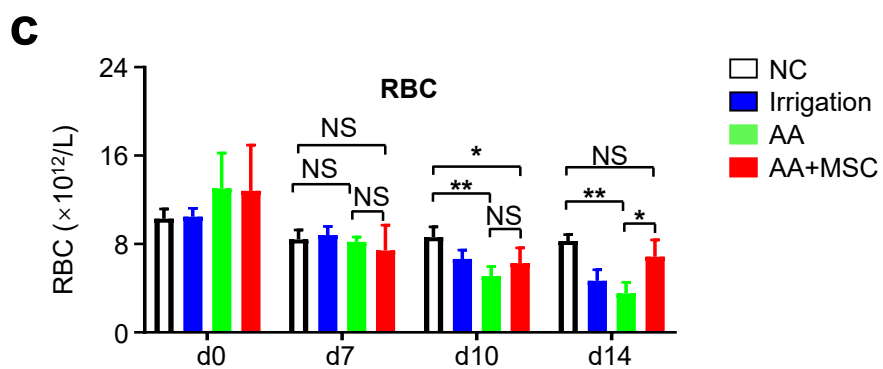

Supplement: Supplementary file 4 — Additional file 4: Figure S4. UC-MSC transplantation ameliorate the pancytopenia in AA mice. [file 13287_2020_1577_MOESM4_ESM.pdf]

**a**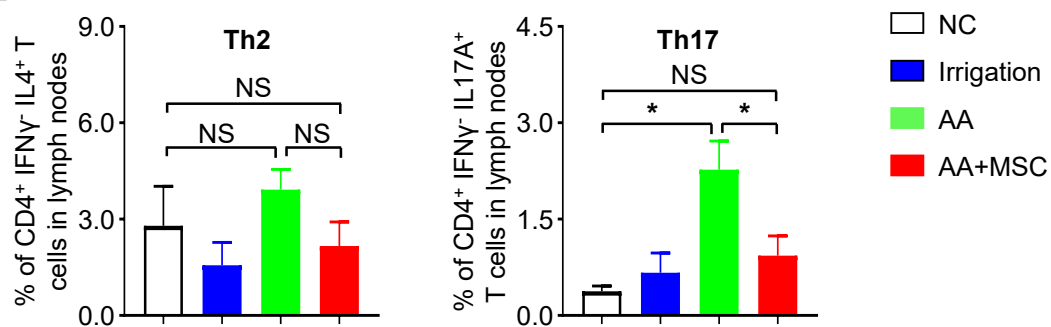**b**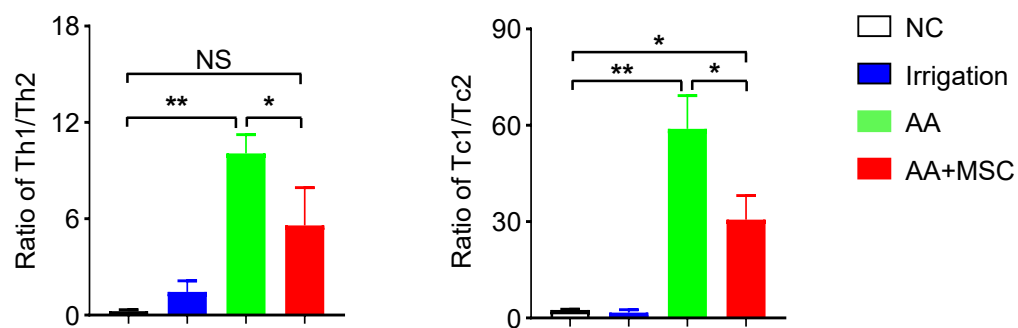

Supplement: Supplementary file 5 — Additional file 5: Figure S5. UC-MSC transplantation significantly rescue the hyperimmune status of AA mice. [file 13287_2020_1577_MOESM5_ESM.pdf]
