# Supplementary material for: Multifaceted characterization of the signatures and efficacy of mesenchymal stem/stromal cells in acquired aplastic anemia
Source: Stem Cell Res Ther. 2020 Feb 13;11:59. doi: 10.1186/s13287-020-1577-2 (PMC7020384; doi:10.1186/s13287-020-1577-2)
Supplement: Supplementary file 11 — Additional file 11: Supplementary information. The details associated with Additional Figure Legends and Additional Tables were listed. [file 13287_2020_1577_MOESM11_ESM.docx]

**Multifaceted Characterization of the Signatures and Efficacy of Mesenchymal Stem/Stromal Cells in Acquired Aplastic Anemia**

Jiali Huo^1^, Leisheng Zhang^1,2*^, Xiang Ren^1^, Xingxin Li^1^, Jinbo Huang^1^, Yingqi Shao^1^, Rucai Zhan^3^, Meili Ge^1^, Zhendong Huang^1^, Jing Zhang^1^, Min Wang^1^, Neng Nie^1^ and Yizhou Zheng^*^

^1^ State Key Laboratory of Experimental Hematology, National Clinical Research Center for Blood Disease, Institute of Hematology & Blood Diseases Hospital, Chinese Academy of Medical Sciences & Peking Union Medical College, Tianjin, 300020, China

^2^ The Postdoctoral Research Station, School of Medicine, Nankai University, Tianjin, 300071, China

^3^ Department of Neurosurgery, The First Affiliated Hospital of Shandong First Medical University, Ji-nan, 250014, China

**Supplemental file 1:**

Supplemental Figure S1-S5;

Supplemental Tables: Table S1-S3.

**Supplementary Figure Legends**

**Figure S1.** The characteristics of AA patients. (**a**) The absolute neutrophil count (ANC) and absolute reticulocyte count (ARC) was significantly decreased in AA patients (HD: n=39; AA: n=49). (**b**) The proportion of Th1 (CD4^+^IFNγ^+^IL4^-^), Th2 (CD4^+^IFNγ^-^IL4^+^), Tc1 (CD8^+^IFNγ^+^IL4^-^) and Tc2 (CD8^+^IFNγ^-^IL4^+^) were detected by flow cytometry. (**c**) There was no significant difference in the concentrate of IL-17A in plasma between AA (n=14) and HD (n=16).

**Figure S2.** The Heatmap of differentially expressed genes between AA (n=3) and HD (n=3). (a) The up-regulated genes in AA. (b) The down-regulated genes in AA.

**Figure S3.** The enriched genetic mutations and variation spectrums in the chromosome of AA-MSCs (HD: n=3; AA: n=3). (**a**) There was no significant difference among the five subtypes of SNPs distribution between AA-MSCs and HD-MSCs. (**b**) The difference of INDELs distribution was also not significant between the two groups. (**c**) The loci regional distribution and expression in the chromosome of gene fusion events (e.g., ATP5I-AP3D1, BLOC1S1-RDH5, CLCF1-POLD4, ACCS-EXT2) were further conformed by the Circos software.

**Figure S4.** UC-MSC transplantation ameliorate the pancytopenia in AA mice. (**a-c**) The complete blood cells analysis revealed significantly increased (**a**) neutrophil, (**b**) reticulocyte and (**c**) red blood cell (RBC) after receiving UC-MSC transplantation (normal mice: n=3; irrigation mice: n=3; AA mice: n=3; AA+MSC: n=3).

**Figure S5.** UC-MSC transplantation significantly rescue the hyperimmune status of AA mice. (**a**) The percentage of Th17 cells rather than Th2 cells was significantly decreased in AA mice after receiving UC-MSC transplantation (normal mice: n=3; irrigation mice: n=3; AA mice: n=3; AA+MSC: n=3). (**b**) Both the ratio of Th1 to Th2 and Tc1 to Tc2 were markedly decreased in the UC-MSC group (normal mice: n=3; irrigation mice: n=3; AA mice: n=3; AA+MSC: n=3).

**Supplementary Tables**

**Table S1. Characteristics of AA patients and healthy donors.**

| # | Diagnosis | Age  (years) | Gender | WBC  (×10^9^/l) | ANC  (×10^9^/l) | Hb  (g/l) | PLT  (×10^9^/l) | ARC  (×10^9^/l) |
| --- | --- | --- | --- | --- | --- | --- | --- | --- |
| 1 | VSAA | 9 | M | 2.43 | 0.38 | 86 | 29 | 53 |
| 2 | VSAA | 22 | M | 1.58 | 0.07 | 67 | 8 | 8 |
| 3 | SAA | 34 | M | 0.81 | 0.01 | 66 | 7 | 4 |
| 4 | VSAA | 14 | F | 1.63 | 0.09 | 61 | 16 | 23 |
| 5 | SAA | 26 | M | 0.9 | 0.34 | 62 | 23 | 8 |
| 6 | VSAA | 51 | F | 1.89 | 0.29 | 84 | 19 | 14 |
| 7 | VSAA | 38 | F | 1.51 | 0.16 | 77 | 22 | 12 |
| 8 | VSAA | 14 | F | 4.25 | 0.15 | 77 | 15 | 4 |
| 9 | VSAA | 13 | M | 1.63 | 0.11 | 95 | 17 | 7 |
| 10 | SAA | 22 | M | 3.13 | 0.05 | 59 | 15 | 17 |
| 11 | VSAA | 10 | F | 3.2 | 0.31 | 47 | 25 | 7 |
| 12 | VSAA | 11 | F | 2.21 | 0.02 | 80 | 10 | 2 |
| 13 | VSAA | 22 | M | 1.59 | 0.44 | 32 | 1 | 6 |
| 14 | SAA | 19 | M | 2.62 | 0.8 | 33 | 5 | 33 |
| 15 | VSAA | 15 | M | 3.17 | 0.15 | 87 | 4 | 9 |
| 16 | SAA | 22 | M | 1.62 | 0.35 | 56 | 10 | 6 |
| 17 | SAA | 37 | M | 2.74 | 0.6 | 71 | 43 | 49.6 |
| 18 | NSAA | 41 | M | 2.98 | 0.73 | 104 | 52 | 52,4 |
| 19 | NSAA | 16 | M | 3.54 | 2.23 | 85 | 45 | 88 |
| 20 | NSAA | 12 | M | 3.13 | 1.33 | 59 | 13 | 75 |
| 21 | NSAA | 26 | F | 3.48 | 1.89 | 124 | 21 | 99.7 |
| 22 | NSAA | 46 | F | 2.04 | 1.18 | 78 | 26 | 51.1 |
| 23 | NSAA | 58 | M | 3.76 | 1.54 | 130 | 12 | 66.8 |
| 24 | NSAA | 44 | M | 3.14 | 1.14 | 145 | 25 | 77.5 |
| 25 | NSAA | 17 | M | 5.55 | 1.64 | 136 | 65 | 62.1 |
| 26 | NSAA | 15 | M | 3.74 | 2.02 | 141 | 58 | 66.9 |
| 27 | NSAA | 44 | M | 4.03 | 2.53 | 59 | 7 | 18.3 |
| 28 | NSAA | 18 | F | 2.34 | 1.01 | 109 | 28 | 78.5 |
| 29 | VSAA | 54 | F | 1.85 | 0.06 | 99 | 11 | 4.3 |
| 30 | SAA | 23 | M | 1.94 | 0.5 | 65 | 27 | 13.3 |
| 31 | VSAA | 15 | M | 1.11 | 0.57 | 77 | 14 | 74.5 |
| 32 | VSAA | 54 | M | 1.39 | 0.08 | 58 | 5 | 1.8 |
| 33 | VSAA | 69 | M | 2.04 | 0.55 | 59 | 31 | 9.2 |
| 34 | SAA | 61 | M | 0.81 | 0.26 | 47 | 23 | 4.6 |
| 35 | VSAA | 48 | M | 0.92 | 0.22 | 58 | 18 | 14.7 |
| 36 | VSAA | 9 | M | 2.48 | 0.22 | 70 | 35 | 19.2 |
| 37 | NSAA | 64 | M | 2.29 | 0.92 | 97 | 16 | 44.9 |
| 38 | VSAA | 36 | M | 1.27 | 0.03 | 64 | 21 | 5.3 |
| 39 | SAA | 36 | M | 1.76 | 0.26 | 69 | 19 | 13.3 |
| 40 | VSAA | 17 | F | 2.45 | 0 | 76 | 9 | 4.8 |
| 41 | VSAA | 55 | M | 1.39 | 0.08 | 58 | 5 | 1.8 |
| 42 | VSAA | 15 | M | 1.65 | 0.07 | 88 | 10 | 4.2 |
| 43 | SAA | 61 | M | 0.81 | 0.26 | 47 | 23 | 4.6 |
| 44 | SAA | 48 | F | 1.93 | 0.37 | 77 | 23 | 17 |
| 45 | SAA | 23 | M | 1.94 | 0.5 | 65 | 27 | 13.3 |
| 46 | SAA | 48 | F | 2.23 | 0.48 | 63 | 6 | 55.8 |
| 47 | NSAA | 13 | M | 3.223 | 0.7 | 88 | 30 | 30.9 |
| 48 | SAA | 31 | F | 1.79 | 0.35 | 90 | 17 | 14.7 |
| 49 | SAA | 19 | M | 1.04 | 0.25 | 64 | 10 | 2.3 |
| 50 | HD | 25 | F | 5.44 | 2.61 | 139 | 257 | 47.5 |
| 51 | HD | 36 | F | 5.98 | 3.77 | 124 | 224 | 91.4 |
| 52 | HD | 27 | M | 6.66 | 3.14 | 135 | 303 | 108.8 |
| 53 | HD | 55 | M | 6.94 | 4.55 | 160 | 261 | 57.0 |
| 54 | HD | 38 | F | 4.44 | 2.38 | 127 | 212 | 66 |
| 55 | HD | 49 | M | 4.36 | 2.35 | 143 | 258 | 148.2 |
| 56 | HD | 37 | M | 8.29 | 4.41 | 157 | 162 | 78.9 |
| 57 | HD | 44 | M | 8.75 | 4.72 | 149 | 293 | 76.9 |
| 58 | HD | 9 | M | 6.2 | 4.05 | 120 | 394 | 83.6 |
| 59 | HD | 47 | M | 5.71 | 2.79 | 160 | 239 | 121.8 |
| 60 | HD | 42 | M | 5.18 | 3.06 | 163 | 200 | 75 |
| 61 | HD | 17 | M | 4.98 | 2.81 | 162 | 278 | 56.3 |
| 62 | HD | 17 | M | 5.97 | 2.67 | 140 | 187 | 80 |
| 63 | HD | 25 | F | 4.54 | 2.72 | 132 | 245 | 58.2 |
| 64 | HD | 17 | M | 5.52 | 2.59 | 157 | 181 | 47.1 |
| 65 | HD | 32 | M | 5.03 | 3.2 | 158 | 273 | 115.3 |
| 66 | HD | 30 | F | 4.45 | 2.37 | 126 | 305 | 61.8 |
| 67 | HD | 32 | M | 7.31 | 4.79 | 125 | 125 | ND |
| 68 | HD | 25 | F | 6.89 | 3.78 | 139 | 245 | 94.6 |
| 69 | HD | 44 | M | 5.99 | 3.08 | 155 | 130 | 82.5 |
| 70 | HD | 30 | F | 6.43 | 4.31 | 128 | 191 | 66.7 |
| 71 | HD | 44 | M | 5.59 | 3.15 | 129 | 118 | 84.8 |
| 72 | HD | 33 | F | 3.68 | 2.07 | 134 | 240 | 42.8 |
| 73 | HD | 53 | F | 6.24 | 3.61 | 147 | 267 | 86 |
| 74 | HD | 41 | F | 5.88 | 3.07 | 136 | 321 | 111.5 |
| 75 | HD | 24 | M | 5.72 | 3.23 | 139 | 368 | 41.1 |
| 76 | HD | 53 | F | 4.59 | 2.76 | 135 | 274 | 51.8 |
| 77 | HD | 23 | F | 9.75 | 5.88 | 135 | 274 | 51.8 |
| 78 | HD | 25 | F | 5.91 | 3.95 | 113 | 232 | 34.9 |
| 79 | HD | 38 | M | 6.83 | 3.81 | 165 | 246 | ND |
| 80 | HD | 29 | F | 6.66 | 4.58 | 164 | 309 | 96 |
| 81 | HD | 28 | M | 6.98 | 4.55 | 154 | 271 | 44.2 |
| 82 | HD | 25 | M | 8.09 | 4.85 | 144 | 178 | 67.1 |
| 83 | HD | 44 | M | 4.95 | 2.89 | 170 | 208 | ND |
| 84 | HD | 53 | M | 6.12 | 3.77 | 175 | 251 | ND |
| 85 | HD | 24 | M | 6.63 | 2.84 | 147 | 291 | 70.1 |
| 86 | HD | 18 | M | 5.94 | 3.81 | 129 | 248 | 48.4 |
| 87 | HD | 43 | F | 4.46 | 2.85 | 128 | 193 | 30.7 |
| 88 | HD | 35 | F | 6.31 | 3.56 | 119 | 263 | 50.3 |

*VSAA: very severe aplastic anemia; SAA: severe aplastic anemia; NSAA: non-severe aplastic anemia; HD: healthy donor; WBC: white blood cell; ANC: absolute neutrophil count; Hb: hemoglobulin; PLT: platelet; ARC: absolute reticulocyte count; ND: not detected; F: female; M: male*

**Table S2. Primers used in this study.**

Real-time PCR primer sequences.

| Gene | Forward sequence (5’→3’) | Reverse sequence (3’→5’) |
| --- | --- | --- |
| *ACTIN* | CTCTTCCAGCCTTCCTTCCT | AGCACTGTGTGTTGGCGTACAG |
| *ADIPOQ* | TGGTCCTAAGGGAGACATCG | TGGAATTTACCAGTGGAGCC |
| *PPAR-γ* | GCTGGCCTCCTTGATGAATA | TGTCTTCAATGGGCTTCACA |
| *RUNX2* | CTCACTACCACACCTACCTG | TCAATATGGTCGCCAAACAGATTC |
| *BGLAP* | GGCGCTACCTGTATCAATGG | TCAGCCAACTCGTCACAGTC |
| *ACAN* | CCCCTGCTATTTCATCGACCC | GACACACGGCTCCACTTGAT |
| *SOX9* | AATGGAGCAGCGAAATCAAC | CAGAGAGATTTAGCACACTGATC |

**Table S3. Antibodies used in this study.**

Antibodies for flow cytometry.

| Antibody |  | Cat. NO. | Source |
| --- | --- | --- | --- |
| Anti-human CD73-PE/Cy7 |  | 344009 | BioLegend |
| Anti-human CD90-FITC |  | 555595 | BD Pharmigen |
| Anti-human CD105-APC |  | 800507 | BioLegend |
| Anti-human CD45-APC |  | 560915 | BD Pharmigen |
| Anti-human CD11b-APC/Cy7 |  | 560914 | BD Pharmigen |
| Anti-human CD34-PE/Cy7 |  | 343515 | BioLegend |
| Anti-human HLA-DR-PE |  | 560943 | BD Pharmigen |
| Anti-human CD4-Percy5.5 |  | 300530 | BioLegend |
| Anti-human CD8-APC |  | 301014 | Biolegend |
| Anti-human CD8-PE/Cy7 |  | 344712 | Biolegend |
| Anti-human CD25-PE |  | 302606 | Biolegend |
| Anti-human CD69-FITC |  | 310924 | Biolegend |
| Anti-human IFNγ-FITC |  | 502506 | Biolegend |
| Anti-human IL-4-PE |  | 12-7049-42 | Invitrogen |
| Anti-human IL17A-APC |  | 17-7149-42 | Invitrogen |
| Anti-mouse CD4-FITC |  | 100510 | Biolegend |
| Anti-mouse CD8-Percy5.5 |  | 100734 | Biolegend |
| Anti-mouse IFNγ-APC |  | 505809 | Biolegend |
| Anti-mouse IL-4-PE |  | 504103 | Biolegend |
| Anti-mouse IL-17A-PE/Cy7 |  | 506921 | Biolegend |
